# 313. Genomic Features Associated with Non-susceptibility to Cefiderocol in Pseudomonas aeruginosa and Cross-resistance to Novel β-lactam/β-lactamase Inhibitors in the Prospective Observational Pseudomonas (POP) study

**DOI:** 10.1093/ofid/ofae631.103

**Published:** 2025-01-29

**Authors:** Stephanie L Egge, Phillip C Thornton, Erika Flores, Rodrigo de Paula Baptista, Karim Clarke, Cesar A Arias, Vincent Tam, Michael J Satlin, William R Miller

**Affiliations:** Oregon Health & Science University, Portland, OR; Houston Methodist Research Institute, Houston, Texas; Houston Methodist Research Institute, Houston, Texas; Houston Methodist Hospital, Houston, Texas; Southwestern University, Georgetown, Texas; Houston Methodist and Weill Cornell Medical College, Houston, TX; University of Houston, Houston, TX; Weill Cornell Medicine, New York, NY; Houston Methodist Research Institute, Houston, Texas

## Abstract

**Background:**

Cefiderocol (FDC) is a last-line agent used to treat carbapenem-resistant P. aeruginosa (CR-PA). We evaluated the prevalence of FDC heteroresistance (hR) and non-susceptibility (NS) in the POP cohort and associations with genes previously related to FDC resistance.

**Figure 1.**
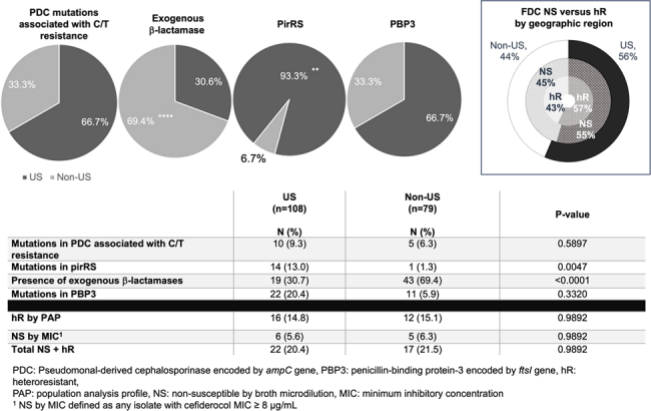
Proportion of isolates harboring genomic feature by geographic region (US vs non-US).

**Methods:**

972 genomes from the multicenter, global POP study were screened for mutations in genes implicated in decreased FDC susceptibility: *ampC*, *pirR*, *pirS*, *pirA*, *piuA/D*, *ftsI*, *cpxS* and exogenous β-lactamases. A representative population was selected to encompass all variants as well as controls obtained by phylogenetically mapping each mutant isolate with a closest non-mutant isolate (n=187). All strains were assessed by broth microdilution (BMD) and population analysis profile (PAP). FDC NS was defined as minimum inhibitory concentration (MIC) ≥ 8 μg/m. hR was determined by PAP area under the curve > 80, per the 99% confidence interval for PAO1. Chi-squared test was used to assess correlation of ceftolozane-tazobactam (C/T), ceftazidime-avibactam (CZA), and imipenem-relebactam (IMR) categorization and FDC hR/NS as well as gene correlations with FDC hR/NS and geographic region of the isolate.

**Figure:**
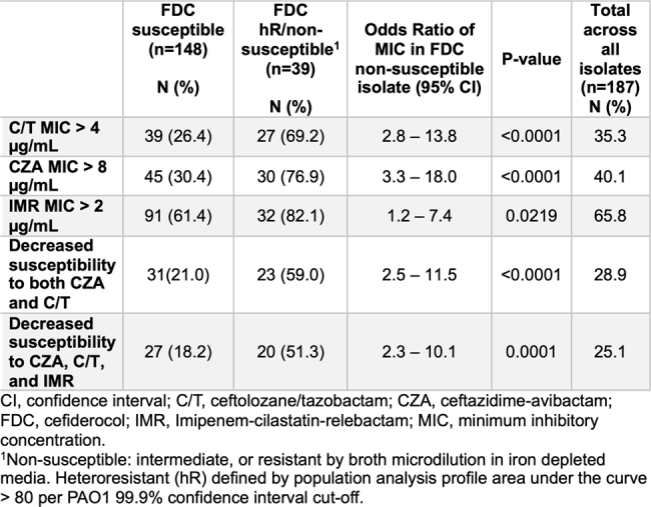

Association between FDC phenotype and MIC category of novel β-lactam/β-lactamase inhibitor combinations.

**Results:**

Over 20% of isolates displayed a NS or hR FDC phenotype (n=39: 28 hR, 11 MIC ≥ 8 μg/mL), equally distributed among US and non-US isolates (**Fig 1**, P =0.36). Isolates with increased C/T, CZA, and IMR MICs were more likely to be FDC NS or hR (**Tbl 1**). IMR susceptibility was low across FDC NS, hR and susceptible cohorts. The combination of mutations in both *ampC* and the PirRS system demonstrated higher risk for FDC NS or hR (**Tbl 2**, P = 0.0003), but isolated mutations in either *ampC* or PirRS did not increase risk for FDC NS or hR. VEB and NDM β-lactamases were present in non-US isolates and their presence correlated with FDC NS or hR (**Tbl 2**, P = 0.0003, 0.0017). Mutations in the PirRS system showed a geographic predisposition in US isolates (**Fig 1**, P= 0.0047), while exogenous β-lactamases were associated with international isolates (P < 0.0001).

**Figure:**
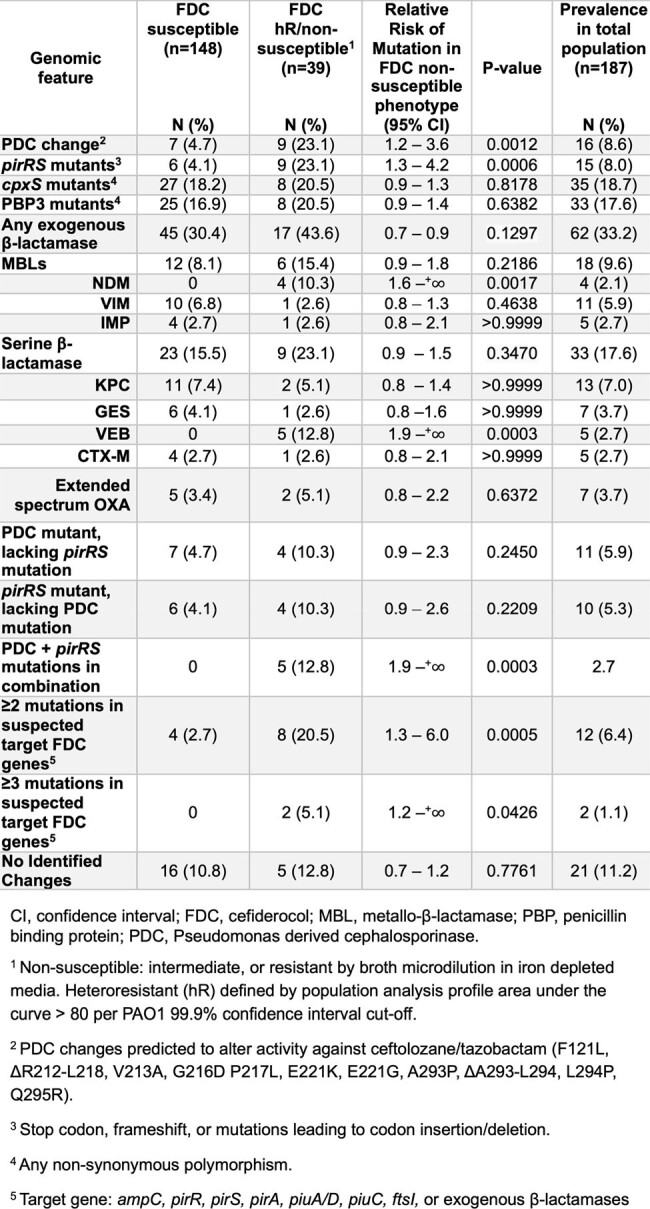

Genotypic determinants associated with FDC susceptibility phenotype.

**Conclusion:**

NS and hR to FDC is prevalent globally among CR-PA and is associated with reduced C/T, CZA, and IMR susceptibility. NS and hR isolates were associated with mutations in *ampC*, *pirRS*, and the exogenous β-lactamases VEB, NDM. Further studies are needed to define clinical significance of FDC hR.

**Disclosures:**

**Cesar A. Arias, MD, MSc, PhD**, UpToDate, Inc.: Royalties **Vincent Tam, Pharm. D.**, AbbVie Inc: Advisor/Consultant **Michael J. Satlin, MD**, AbbVie: DSMB participant|bioMerieux: Grant/Research Support|Merck: Grant/Research Support|Selux Diagnostics: Grant/Research Support|SNIPRBiome: Grant/Research Support **William R. Miller, M.D.**, Merck: Grant/Research Support|UptoDate: Royalties

